# Recent Machine Learning Progress in Lower Limb Running Biomechanics With Wearable Technology: A Systematic Review

**DOI:** 10.3389/fnbot.2022.913052

**Published:** 2022-06-02

**Authors:** Liangliang Xiang, Alan Wang, Yaodong Gu, Liang Zhao, Vickie Shim, Justin Fernandez

**Affiliations:** ^1^Faculty of Sports Science, Ningbo University, Ningbo, China; ^2^Research Academy of Grand Health, Ningbo University, Ningbo, China; ^3^Auckland Bioengineering Institute, The University of Auckland, Auckland, New Zealand; ^4^Faculty of Medical and Health Sciences, The University of Auckland, Auckland, New Zealand; ^5^Department of Engineering Science, Faculty of Engineering, The University of Auckland, Auckland, New Zealand

**Keywords:** gait, wearable sensor, machine learning, deep learning, running, lower limb

## Abstract

With the emergence of wearable technology and machine learning approaches, gait monitoring in real-time is attracting interest from the sports biomechanics community. This study presents a systematic review of machine learning approaches in running biomechanics using wearable sensors. Electronic databases were retrieved in PubMed, Web of Science, SPORTDiscus, Scopus, IEEE Xplore, and ScienceDirect. A total of 4,068 articles were identified *via* electronic databases. Twenty-four articles that met the eligibility criteria after article screening were included in this systematic review. The range of quality scores of the included studies is from 0.78 to 1.00, with 40% of articles recruiting participant numbers between 20 and 50. The number of inertial measurement unit (IMU) placed on the lower limbs varied from 1 to 5, mainly in the pelvis, thigh, distal tibia, and foot. Deep learning algorithms occupied 57% of total machine learning approaches. Convolutional neural networks (CNN) were the most frequently used deep learning algorithm. However, the validation process for machine learning models was lacking in some studies and should be given more attention in future research. The deep learning model combining multiple CNN and recurrent neural networks (RNN) was observed to extract different running features from the wearable sensors and presents a growing trend in running biomechanics.

## Introduction

Machine learning approaches have been widely utilized in gait biomechanics studies in the past decades (Ferber et al., 2016; Halilaj et al., 2018; Xiang et al., 2022a). Most runners frequently suffer running-related injuries in the lower limb and foot (van Gent et al., 2007; Murr and Pierce, 2019). Based on learning-driven sensor data, machine learning and deep learning could provide gait monitoring in real-time, recommendations for running shoes (Koska and Maiwald, 2020; Young et al., 2020), and suggestions for running injury protection (Girka et al., 2020; Matijevich et al., 2020; Dempster et al., 2021). It is crucial to know how machine learning techniques are implemented in lower limb running biomechanics by exploring wearable sensor data.

Traditional gait biomechanical analysis methods use statistical hypothesis tests based on discrete variables and summary metrics, such as the mean peak angles (Taylor et al., 2013; Halilaj et al., 2018; Dixon et al., 2019). *A priori* selection of features and sufficient background knowledge are essential to conducting further analysis and that may decrease the objectivity (Phinyomark et al., 2015, 2018). Consequently, these statistical tools cannot explain the complexity of multi-variables and process data-intense tasks (Halilaj et al., 2018). In the era of big data, machine learning as a growing data science method can process and analyze the large amounts of gait biomechanics data, for instance, wearable sensor data, and achieve state-of-the-art performance (Ferber et al., 2016).

Machine learning incorporates supervised learning and unsupervised learning. In the realm of biomechanics, it has been utilized for dimensionality reduction of high-dimensional data (Phinyomark et al., 2015; Watari et al., 2018a), gait classification (Fukuchi et al., 2011; Taylor et al., 2013; Clermont et al., 2017), pathology detection (Bennetts et al., 2013; Christian et al., 2016; Li et al., 2020), and human activity recognition (Ordóñez and Roggen, 2016; Ignatov, 2018; Ihianle et al., 2020). Principle component analysis (PCA) as an unsupervised learning method is commonly used for feature extraction before training (Wu and Wang, 2008; Taylor et al., 2013; Phinyomark et al., 2014; Clermont et al., 2017; Mei et al., 2020; Suda et al., 2020). Clermont et al. (2017) classified competitive and recreational runners based on lower limb kinematics data using the support vector machine (SVM). Machine learning algorithms can also discriminate runners' experience level and gender *via* assessing running's spatiotemporal parameters (Clermont et al., 2019b). Bennetts et al. (2013) identified the typical peak plantar pressure distributions utilizing k-means clustering.

Lower limb gait biomechanical studies are typically limited to the laboratory-based setting (Xiang et al., 2022b), such as measuring knee joint angles and moments using an optical motion capture system and force plates (Liu et al., 2020). Inertial measurement unit (IMU) is portable, lightweight, and low-cost, and can be used in an unconstrained environment (Fong and Chan, 2010; Ahamed et al., 2019). Wearable sensors have gained popularity for gait analysis in recent years. A recent systematic review from Camomilla et al. (2018) summarized the growing trend of utilizing wearable inertial sensors in the field of biomechanical analysis and activity classification. Picerno (2017) compared different approaches to evaluating lower limb joint kinematics by using IMU sensors. Deep learning is a subset of machine learning algorithms based on artificial neural networks (ANN). In recent years, it has been widely used in computer vision (Lee et al., 2009; Sermanet et al., 2013), speech recognition (Sainath et al., 2015), and medical image analysis (Ker et al., 2017; Shen et al., 2017). Deep neural network structures have advantages in processing time-series sensor data and require lower computational cost than traditional machine learning approaches. Integrated with deep learning algorithms, wearable sensors can be utilized for pattern recognition (Cust et al., 2019) and biomechanical variable prediction (Stetter et al., 2019; Hernandez et al., 2021) without experiment and environmental limitations.

Hu et al. (2018) found that long short-term memory (LSTM) recurrent neural networks (RNN) can detect surface- and age-related differences in walking gait based on a single wearable IMU sensor. Ronao and Cho (2016) showed that the accelerometer and gyroscope sensor data adopting deep convolutional neural networks (CNN) achieved high accuracy for human activity recognition. Ordóñez and Roggen (2016) presented a deep learning framework of the convolutional and LSTM (DeepConvLSTM) for accurate human activity recognition. Convolutional layers act as feature extractors capturing spatial domain features from one-dimensional sensor data, while recurrent layers are used to extract temporal domain features (Hernandez et al., 2021). The performance improves by fusing the accelerometer, gyroscope, and magnetic field sensors compared to the acceleration or acceleration and angular velocity data (Ordóñez and Roggen, 2016).

IMU sensors are heavily used technologies in distance running (Zrenner et al., 2020). Using inertial sensor-based data during running, one can classify different conditions and predict kinetical variables (Clermont et al., 2019b; Pogson et al., 2020). Jogging gait phase and period were detected and identified by lower extremity placed accelerometers and gyroscopes using machine learning algorithms (Mannini and Sabatini, 2012; Zdravevski et al., 2017). Kobsar et al. (2014) utilized PCA to classify training background from running experience. In a study by Pogson et al. (2020), PCA and multilayer perceptron (MLP) were adopted to predict ground reaction force (GRF) from trunk acceleration. Running speed conditions and running environments were assessed and classified using SVM with an IMU sensor placed on the participants' lower back during data collection (Benson et al., 2018b, 2020).

In 2018, Benson et al. (2018a) reviewed the progress of IMU sensors in gait analysis. O'Reilly et al. (2018) evaluated the lower limb exercise detection accuracy of the wearable inertial sensor. However, that review retrieved articles till 2017 and did not include running gait. A systematic review from Farrahi et al. (2019) revealed that machine learning techniques could predict activity type and intensity based on raw acceleration data. Fong and Chan (2010) estimated the use of wearable sensors in lower limb biomechanics. The approaches to assessing lower limb joint kinematics by using wearable sensors have been summarized by Picerno (2017). A systematic review in 2019 illustrated wearable inertial sensors' performance in sport-specific movement recognition using machine learning and deep learning approaches (Rapp et al., 2021). Although there is a growing trend concerning machine learning in lower limb running biomechanics, particularly deep learning algorithms in wearable inertial sensor studies, there are few studies on the accuracy of machine learning approaches as utilized in lower limb running biomechanics integrating wearable inertial sensors. Furthermore, no compelling evidence illustrated the application scenes of different machine learning algorithms, the requirements of sensor placement based on the research goal, and how the model was assessed and validated in lower limb running biomechanics.

Based on the currently existing knowledge gap, the purpose of this initial study is to conduct a systematic review regarding machine learning and deep learning approaches used in running biomechanics and was limited to the wearable sensors placed in lower limbs. By investigating the eligible studies, we hope: (1) to elaborate on different kinds of machine learning techniques used in running biomechanics and its performance; (2) to recommend suitable sensor placement locations to obtain decent accelerations or angular velocities or other biomechanical variables; (3) improve predictive accuracy for the related studies in gait analysis in the future.

## Methods

### Search Strategy

**Figure 1:**
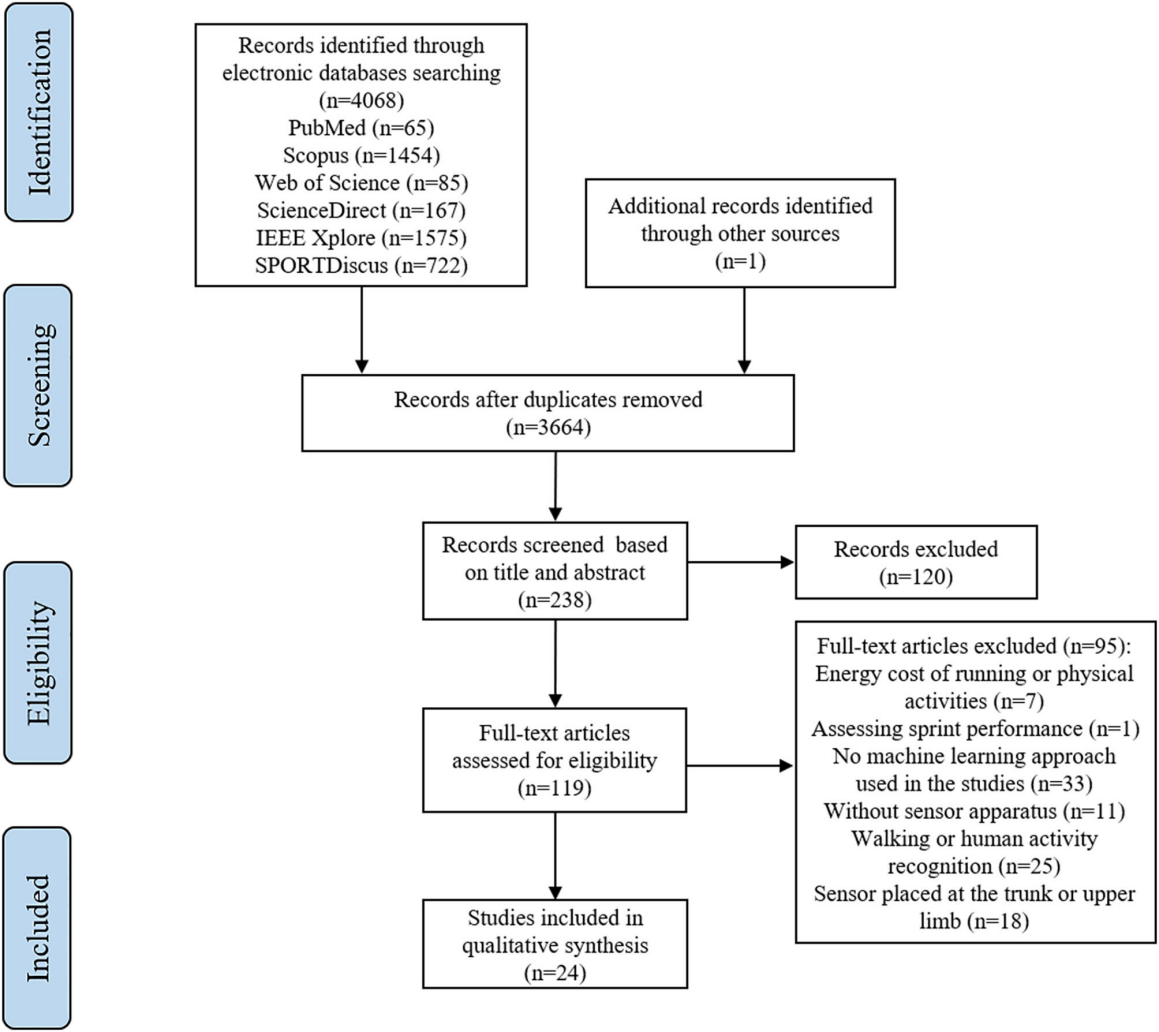
PRISMA flow diagram for original research articles' searching and screening process.

This systematic review followed the PRISMA (Preferred Reporting Items for Systematic Reviews and Meta-Analyses) recommendations (Moher et al., 2009). The protocol for this systematic review was registered on INPLASY (NO. 202210083). Electronic databases were retrieved in PubMed, Web of Science, SPORTDiscus, Scopus, IEEE Xplore, and ScienceDirect by one reviewer (L.X.) to identify original research articles published up to May 2021 (from 2000). The paper screening was conducted by two investigators independently (L.X. and A.W.). After an initial search, relative article information was input to Rayyan QCRI (Ouzzani et al., 2016) for duplicate removal, study screening, and identification. A backward search was conducted on the studies included. One study was added from the reference lists of included studies to review the flow for further paper screening. Non-English articles and conference proceedings, and dissertations were excluded. The retrieve strategy and limit conditions are shown in Table 1. Four different categories were used to identify relevant studies: wearable inertial sensor, machine learning and deep learning, lower limb, and running. By using Boolean operation, the retrieved studies at least contain one keyword in the full field. The flow diagram of the paper search and screen process is presented in Figure 1.

**Table 1:** Electronic databases retrieve strategy.

**Search items**	**Limit conditions**
**PubMed, Web of Science, SPORTDiscus, Scopus**
(“wearable sensor” OR “inertial sensor” OR “accelerometer” OR “gyroscope” OR “IMU”) AND (“machine learning” OR “classification” OR “regression” OR “clustering” OR “PCA” OR “SVM” OR “KNN” OR “decision tree” OR “boosting” OR “random forest” OR “deep learning” OR “neural network*” OR “CNN” OR “RNN” OR “LSTM” OR “ConvLSTM” OR “DeepConvLSTM”) AND (“running” OR “jogging”) AND (“gait” OR “lower limb” OR “lower extremity” OR “plantar pressure” OR “foot” OR “ankle” OR “shank” OR “knee” OR “thigh”)	Keywords in all field of the article; Advanced search; Article type: Journal; Language: English; Publish time: From 2000 to May 2021
**IEEE Xplore**
(“wearable sensor” OR “inertial sensor” OR “IMU”) AND (“machine learning” OR “classification” OR “regression” OR “clustering” OR “deep learning” OR “neural network*”) AND (“running” OR “jogging”) AND (“gait” OR “lower limb” OR “lower extremity”)	Keywords in all field of the article; Advanced search; Article type: Journal; Language: English; Publish time: From 2000 to May 2021
**ScienceDirect**
(“wearable sensor” OR “inertial sensor” OR “IMU”) AND (“machine learning” OR “deep learning”) AND (“running”) AND (“gait” OR “lower limb”)	Keywords in full text and metadata; Advanced search; Article type: Journal; Language: English; Publish time: From 2000 to May 2021

### Eligibility Criteria

Articles were selected based on the PICO principle (participants, intervention, comparisons, and outcomes). Our research identified studies that utilized machine learning and deep learning approaches and wearable inertial sensors to assess running biomechanics. For the included studies, wearable sensors must be placed in the participants' lower limbs. Only inertial sensors were selected in this review, including accelerometer, gyroscope, and magnetometer. Studies were removed if the statistical regression model rather than the machine learning approach was employed for the prediction. Energy expenditure and sprint performance assessment studies were not considered in this study. Studies using machine learning and wearable sensors only evaluating walking gait and human activity recognition were excluded.

### Quality Assessment

The methodological quality of the included studies was assessed by the modified QualSyst Assessment Tool for quantitative studies (Kmet et al., 2004). A total of ten items were identified in this scale for aspects of the research question, study design, statistical analysis, sample size, and results reporting. Each item was scored as “yes” = 2, “partial” = 1, or “no” = 0 given the degree of the specific criteria that were met. The risk of bias was initially evaluated by three investigators (L.X., A.W., and J.F.), independently and confirmed by two reviewers (A.W. and J.F.). Each study's score was calculated by summing 10 items' scores and dividing by the total score (Kandula et al., 2016; Capiau et al., 2020). The evaluation questions include If the question or objective is clearly described? Is design evident and appropriate to answer the study question? If the method of subject selection or source of information/input variables is described and appropriate? Is the subject's characteristics, input variables, or information sufficiently described? Is the outcome well defined and robust to measurement/misclassification bias? If means of assessment are reported? Is the sample size appropriate? If analysis is described and appropriate? If some estimate of variance is reported for the main results? Are the results reported in sufficient detail? Do the results support the conclusions?

### Data Extraction

Information was extracted from the included 24 studies based on participants and sensor characteristics, and machine learning approaches. Participant information included participant numbers, gender, age, type, and running speed during the data collection. Sensor characteristics contained sensor number, placement in the low limb, brand, and sampling frequency for data collection. For machine learning and deep learning, specific approaches, percentage of training and test dataset, cross-validation (CV) methods, data preprocessing, predictors, response, ground true reference, evaluation, and performance were extracted from the included literature.

## Results

### Search Results

A total of 4,068 articles were identified *via* the electronic databases retrieve, and one additional study was found from the reference lists. Then, 405 duplicate articles were removed; 119 studies remained for the full-text evaluation after the screening of the study's title and abstract; 95 articles were excluded based on the exclusion criteria. Finally, 24 articles were included in this systematic review.

### Quality Assessment

As shown in Table 2, the range of quality scores of the included studies is from 0.78 to 1.00, and the mean score is 0.91. The main sources of bias are the small sample size and unclear subject characteristics. Seven studies vaguely reported the study design. Statistical analysis was not included in eight studies, but machine learning approaches' performance and accuracy were reported.

**Table 2:** Methodological quality assessment by the modified QualSyst quality appraisal tool.

	**Q1**	**Q2**	**Q3**	**Q4**	**Q5**	**Q6**	**Q7**	**Q8**	**Q9**	**Q10**	**Total**	**Summary score**
Stetter et al. (2020)	+2	+1	+1	+1	+2	+1	+2	+2	+2	+2	16	0.80
Stetter et al. (2019)	+2	+1	+2	+1	+2	+1	+2	+2	+2	+2	17	0.85
Hernandez et al. (2021)	+2	+1	+2	+2	+2	+2	+2	+2	+2	+2	19	0.95
Gholami et al. (2020)	+2	+2	+2	+1	+2	+1	N/A	+1	+2	+2	15	0.83
Wouda et al. (2018)	+2	+2	+2	+2	+2	+1	+2	+2	+2	+2	19	0.95
Derie et al. (2020)	+2	+1	+2	+2	+2	+2	+2	+2	+2	+2	19	0.95
Liu et al. (2020)	+2	+1	+2	+2	+2	+1	+2	+2	+1	+2	17	0.85
Rapp et al. (2021)	+2	+2	+2	+1	+2	+2	+2	+2	+2	+2	19	0.95
Ngoh et al. (2018)	+2	+2	+2	+1	+2	+1	N/A	+2	+2	+2	16	0.89
Young et al. (2020)	2	2	1	1	2	2	N/A	1	1	2	14	0.78
Robberechts et al. (2021)	+2	+2	+2	+2	+2	+2	+2	+2	+2	+2	20	1.00
Zrenner et al. (2018)	+2	+2	+2	+1	+2	+2	N/A	+2	+2	+2	17	0.94
Komaris et al. (2019)	+1	+1	+2	+2	+2	+2	+2	+2	+2	+2	18	0.90
Tan et al. (2019)	+2	+1	+2	+1	+2	+1	N/A	+1	+2	+2	14	0.78
Watari et al. (2018a)	+2	+2	+2	+2	+2	+1	+2	+2	+1	+2	18	0.90
Watari et al. (2018b)	+2	+2	+2	+2	+2	+1	+2	+2	+2	+2	19	0.95
Ahamed et al. (2019)	+2	+2	+2	+1	+2	+1	+2	+2	+2	+2	18	0.90
Ahamed et al. (2018)	+2	+2	+2	+2	+2	+1	+2	+2	+2	+2	19	0.95
Clermont et al. (2019a)	+2	+2	+2	+2	+2	+1	+2	+2	+2	+2	19	0.95
Dixon et al. (2019)	+2	+2	+2	+2	+2	+2	N/A	+2	+2	+2	18	1.00
Johnson et al. (2021)	+2	+2	+2	+1	+2	+2	N/A	+2	+2	+2	17	0.94
Tan et al. (2020)	+2	+2	+2	+1	+2	+1	+2	+2	+2	+2	18	0.90
Koska and Maiwald (2020)	+2	+2	+2	+2	+2	+1	N/A	+2	+2	+2	17	0.94
Matijevich et al. (2020)	+2	+2	+2	+1	+2	+1	+2	+2	+2	+2	18	0.90
												0.91

*2 = “yes”, 1 = “partial”, 0 = “no”. N/A means that study should not be checked for this question. Summary score = total sum/total possible sum*.

*Q1: Question or objective clearly described?*

*Q2: Design evident and appropriate to answer the study question?*

*Q3: Method of subject selection or source of information/input variables is described and appropriate*.

*Q4: Subject characteristics or input variables/information sufficiently described?*

*Q5: Outcome well defined and robust to measurement/misclassification bias? Means of assessment reported?*

*Q6: Sample size appropriate?*

*Q7: Analysis described and appropriate?*

*Q8: Some estimate of variance is reported for the main results?*

*Q9: Results reported in sufficient detail?*

*Q10: Do the results support the conclusions?*

### Participants' Characteristics

**Figure 2:**
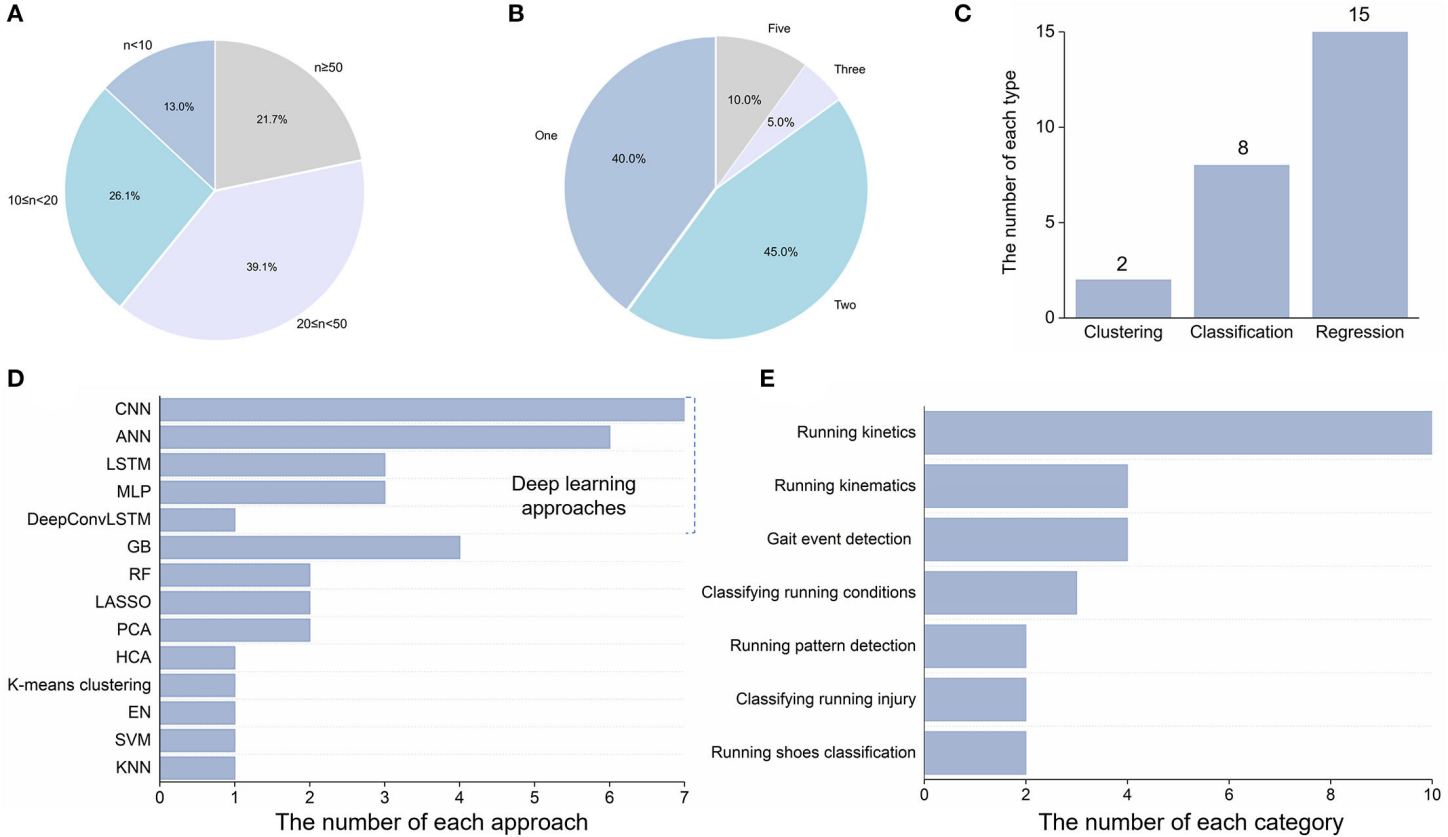
Characteristic information: **(A)** sample size; **(B)** the number of sensors; **(C)** types of machine learning algorithms; **(D)** machine learning approaches; **(E)** purpose of machine learning.

The majority of participants were healthy subjects, except for three studies that contained patellofemoral pain participants or subjects with running-related injuries (Table 3) (Watari et al., 2018a,b; Rapp et al., 2021). The healthy subjects included both experienced runners and novice runners. The sample size was from 6 to 580, and three articles with a sample size below ten (Figure 2A) (Ahamed et al., 2018; Ngoh et al., 2018; Wouda et al., 2018). Approximately 40% of articles recruited participants with numbers between 20 and 50. In six studies, only male subjects were considered (Ngoh et al., 2018; Wouda et al., 2018; Stetter et al., 2019, 2020; Gholami et al., 2020; Hernandez et al., 2021). Age characteristics in three articles were not reported (Young et al., 2020; Johnson et al., 2021; Rapp et al., 2021). Several studies tested various speeds, except the running speed not mentioned in two studies (Dixon et al., 2019; Tan et al., 2019).

**Table 3:** Participants and wearable inertial sensor specifications.

**References**	**Participants (male/female)**	**Age (years)**	**Participant characteristics**	**Running speed**	**Number of sensors**	**Sensor placement**	**Sensor brand**	**Sampling frequency**	**Accelerometer**	**Gyroscope**	**Magnetometer**
Stetter et al. (2020)	13 (13/0)	26.1 ± 2.9	Healthy subjects	Moderate running, fast running (speed not mentioned)	2	Right thigh and shank	Custom-built IMUs	1,500 Hz	Tri-axis; range: ±8 g	Tri-axis; range: ±2,000/s	/
Stetter et al. (2019)	13 (13/0)	26.1 ± 2.9	Healthy subjects	Moderate running, fast running (speed not mentioned)	2	Right thigh and shank	Custom-built IMUs	1500 Hz	Tri-axis; range: ±8 g	Tri-axis; range: ±2,000/s	/
Hernandez et al. (2021)	27 (27/0)	26.5 ± 3.9	Healthy subjects	8–14 km/h	5	Pelvis, left and right thigh and tibias	PUSH Pro system	100 Hz	Tri-axis	Tri-axis	/
Gholami et al. (2020)	10 (10/0)	27.0 ± 4.0	Healthy subjects	8–12 km/h	1	On the shoes (dorsum)	Xsens (MTw Awinda)	100 Hz	Tri-axis	Tri-axis	Tri-axis
Wouda et al. (2018)	8 (8/0)	25.1 ± 5.2	Experienced runners	10,12, 14 km/h	3	Pelvis and lower legs	Xsens	240 Hz	Tri-axis	Tri-axis	Tri-axis
Derie et al. (2020)	93 (55/38)	35.3 ± 0.9	Recreational and competitive rear foot runners	2.55, 3.2, 5.1 m/s and preferred running speed	2	Left and right tibias	LIS331, Sparfkun	1,000 Hz	Tri-axis	/	/
Liu et al. (2020)	30 (16/14)	31.6 ± 3.2	Competitive, recreational and novice runners	7–17 km/h	2	Left and right distal tibias	MyoMOTION (Noraxon)	200 Hz	Tri-axis; range: ±16 g	Tri-axis; range: ±2,000/s	Tri-axis; range: ±1.9 Gauss
Rapp et al. (2021)	580 (292/288)	NR	Healthy participants and subjects with running-related lower limb injuries	Self-selected speeds	/	Sacrum, left and right thighs, left and right shanks, and left and right feet	Virtual IMUs	/	Tri-axis	Tri-axis	/
Ngoh et al. (2018)	7 (7/0)	21.3 ± 0.5	Healthy subjects	8–10 km/h	1	Right running shoe (above the third metatarsal)	Opal inertial sensor (APDM Inc.)	NR	Tri-axis; range: ±6 g	Tri-axis; range: ±2,000/s	Tri-axis; range: ±6 Gauss
Young et al. (2020)	203 (91/112)	NR	Healthy subjects	8 km/h	2	Left and right foot	MYMO	60 Hz	Tri-axis	Tri-axis	/
Robberechts et al. (2021)	93 (55/38)	35.3 ± 0.9	Rearfoot runners	2.55, 3.2, 5.1 m/s and preferred running speed	2	Left and right shins	LIS331, Sparkfun	1,000 Hz	Tri-axis	/	/
Zrenner et al. (2018)	27 (21/6)	24.9 ± 2.4	Amateur runners (forefoot/midfoot runners: 6, rearfoot runners: 21)	2–6 m/s	2	Left and right shoes midsole	miPod	200 Hz	range: ±16 g	Range: ±2,000/s	/
Komaris et al. (2019)	28 (27/1)	34.8 ± 6.6	Competitive or elite runners	2.5, 3.5, and 4.5 m/s	/	Left and right shank	Virtual accelerometer	/	Tri-axis	/	/
Tan et al. (2019)	20 (12/8)	33.4 ± 7.0	Healthy subjects	Running speed not mentioned (including indoor run, treadmill run, outdoor run)	2	Left and right ankle	Shimmer3	128 Hz	Tri-axis; range: ±8 g	/	/
Watari et al. (2018a)	41 (29/12)	30.8 ± 3.2	Runners with patellofemoral pain	2.7 m/s	/	Pelvic	Virtual accelerometer	/	Tri-axis	/	/
Watari et al. (2018b)	110 (44/66)	34.1 ± 2.9	Runners with patellofemoral pain	2.61 ± 0.2 m/s	/	Pelvic	Virtual accelerometer	/	Tri-axis	/	/
Ahamed et al. (2019)	11 (10/1)	37.3 ± 11.7	Recreational runners	2.35 ± 0.1 m/s	1	Pelvic	Lumo Run	100 Hz	Tri-axis	Tri-axis	Tri-axis
Ahamed et al. (2018)	6 (5/1)	38.3 ± 13.1	Recreational runners	2.18–2.54 m/s	1	Pelvic	Lumo Run	100 Hz	Tri-axis	Tri-axis	Tri-axis
Clermont et al. (2019a)	27 (12/15)	45.7 ± 6.7	Marathon runners	8.56–9.55 km/h	1	Pelvic	Lumo Run	100Hz	Tri-axis	Tri-axis	Tri-axis
Dixon et al. (2019)	29 (15/14)	23.3 ± 3.6	Untrained subjects (*n* = 10), recreational (*n* = 9), and well-trained (*n* = 10) runners	NR	1	Right tibia	X50-2, Gulf coast data concepts	1,024 Hz	Tri-axis; range: ±50 g	/	/
Johnson et al. (2021)	Training dataset: NR (male: 59.9%, female: 40.1%); test dataset: 5 (4/1)	NR	Training dataset: young adult athletes, test dataset: team-sport athletes	Slow speed running (2–3 m/s), moderate speed running (4–5 m/s), and fast speed running (>6 m/s)	5	Pelvis, bilateral thigh, bilateral shank	Noraxon DTS-3D 518 (test dataset only)	NR	Tri-axis	/	/
Tan et al. (2020)	15 (8/7)	23.9 ± 1.1	Recreational runners	2.4 and 2.8 m/s	1	Left shank	MTi-300, Xsens	200 Hz	Tri-axis	Tri-axis	NR
Koska and Maiwald (2020)	22 (10/12)	29 ± 5.9	Recreational runners	10.7 ± 0.7 km/h	1	Heel cup of the left running shoe	InvenSense ICM-20601	2,000 Hz	Tri-axis; range: ±32 g	Tri-axis; range: ± 4,000/s	/
Matijevich et al. (2020)	10 (5/5)	24 ± 2.5	Recreational runners	2.6–4.0 m/s	2	Shank and foot	Virtual IMUs	/	NR	/	/

*NR, not reported in the study; IMUs, inertial measurement units*.

### Sensor Information

The number of IMUs sensors placed on the lower limbs was 1, 2, 3, and 5 (as shown in Figure 2B). Pelvis, thigh, shank, distal tibia, and foot were frequently selected as the placement location. Sensor(s) was placed on or in the running shoes in four studies (Ngoh et al., 2018; Zrenner et al., 2018; Gholami et al., 2020; Koska and Maiwald, 2020). Three types of IMUs sensors are utilized, including commercial sensors, custom-built sensors, and virtual sensors. All studies contain acceleration data with the range of accelerometers from ±6 to ±50 g. Gyroscope data were not contained in nine articles (Watari et al., 2018a,b; Dixon et al., 2019; Komaris et al., 2019; Tan et al., 2019; Derie et al., 2020; Matijevich et al., 2020; Johnson et al., 2021; Robberechts et al., 2021) and for most of the studies, magnetometer data were not incorporated in the IMU sensor (Watari et al., 2018a,b; Zrenner et al., 2018; Dixon et al., 2019; Komaris et al., 2019; Stetter et al., 2019, 2020; Tan et al., 2019; Derie et al., 2020; Koska and Maiwald, 2020; Matijevich et al., 2020; Young et al., 2020; Hernandez et al., 2021; Johnson et al., 2021; Rapp et al., 2021; Robberechts et al., 2021).

### Machine Learning and Deep Learning

#### Approaches and Purposes

As depicted in Figures 2C–E, Deep learning algorithms occupied 57% of total machine learning approaches, compared with 43% of traditional machine learning algorithms. CNN was the most frequently used algorithm (seven times), followed by ANN and long short-term memory (LSTM). The DeepConvLSTM model was recently applied to lower limb joint prediction from linear acceleration and angular velocity data of the IMUs sensor (Hernandez et al., 2021). Gradient boosting (GB) algorithm included gradient boosting regression tree (Derie et al., 2020) and decision tree (Dixon et al., 2019; Liu et al., 2020; Young et al., 2020). There are two unsupervised learning algorithms [i.e., hierarchical cluster analysis (Watari et al., 2018b) and K-means clustering (Koska and Maiwald, 2020)] adopted for the clustering tasks. Eight machine learning approaches were used to classify different conditions. Regression tasks were the primary intention in 15 articles.

Lower limb running kinematics and kinetics were predicted using wearable inertial sensors combined with regression algorithms, including joint angles, forces, and moments and GRF (as shown in Table 4). Different types of outdoor terrain (Dixon et al., 2019), inclinations of the running surface (Ahamed et al., 2019), and environmental weather conditions (Ahamed et al., 2018) were detected and classified in three studies. The accuracy of gait event and spatiotemporal parameter detections was also tested (Zrenner et al., 2018; Tan et al., 2019; Liu et al., 2020; Robberechts et al., 2021). Two studies aimed at the running pattern or level classification (Clermont et al., 2019a; Liu et al., 2020). One study divided patellofemoral pain patients into two subgroups based on running gait (Watari et al., 2018b). The accuracy of identifying the effectiveness of exercise treatment for patellofemoral pain patients was evaluated with an SVM classifier by the same team (Watari et al., 2018a). PCA was adopted for dimensionality reduction in these two articles. According to foot posture and foot strike pattern, the ensemble deep learning model could accurately assess and recommend running shoe types to runners with an average accuracy of 94.6% (Young et al., 2020). Comfortable and uncomfortable running shoes also could be identified from the heel's sagittal plane angular velocity data using the k-nearest neighbors' classifier (KNN) (Koska and Maiwald, 2020).

**Table 4:** The detailed machine learning approaches.

**References**	**Predictor**	**Data preprocessing**	**Dataset split**	**CV**	**Machine learning approaches**	**Response**	**Evaluation**	**Performance**	**Ground truth/** **biomechanical model reference data**
Stetter et al. (2020)	Tri-axial linear acceleration and tri-axial angular velocity	Filtered by a 4th order Butterworth filter with cut-off frequency of 15 Hz; IMUs signals were interpolated to keep the same sample frequency with knee joints moments data	/	LOSOCV	ANN (two hidden layers, 100 and 20 neurons)	External knee flexion and adduction moments	*R*^2^, rRMSE, RMSE	Knee flexion moment: Moderate running: *R*^2^ = 0.85, RMSE = 0.58 Nm/kg, rRMSE = 19.7%; fast running: *R*^2^ = 0.65, RMSE = 1.13 Nm/kg, rRMSE = 25.5%. Knee adduction moment: Moderate running: *R*^2^ = 0.4, RMSE = 0.37 Nm/kg, rRMSE = 34.4%; fast running: *R*^2^ = 0.21, RMSE = 0.8 Nm/kg, rRMSE = 33.8%	Kinematics and kinetics were collected by using a Vicon motion capture system and two AMTI plates simultaneously. Knee flexion and adduction moments were calculated *via* an inverse dynamic modeling
Stetter et al. (2019)	Tri-axial linear acceleration and tri-axial angular velocity	Filtered by a 4th order Butterworth filter with cut-off frequency of 15 Hz; IMUs signals were interpolated to keep the same sample frequency with knee joints forces data	Training/validation/ test: 0.70/0.15/0.15	LOSOCV	ANN (two hidden layers, 250 and 100 neurons)	Vertical, anterior-posterior, and medial-lateral knee joint forces	*R*^2^, rRMSE	Moderate running: mean *R*^2^ = 0.76, mean rRMSE = 25%; fast running: mean *R*^2^ = 0.73, mean rRMSE = 28.7%	Kinematics and kinetics were collected by using a Vicon motion capture system and two AMTI plates simultaneously. Knee joint forces were calculated *via* an inverse dynamic modeling
Hernandez et al. (2021)	Tri-axial linear acceleration and tri-axial angular velocity	Data were standardized using Z-score normalization	Training/ validation/test: 19/4/4 subjects	Nested k-fold CV (user-independent approach)	DeepConvLSTM (two convolutional layers, two recurrent layers, sliding window: 100, step size:100)	Lumbar extension, bending, and rotation; hip flexion, adduction, and rotation (left and right); knee flexion (left and right); ankle dorsiflexion and inversion (left and right)	*R*^2^, ME, MAE	Mean *R*^2^ = 0.9 ± 0.16, mean MAE = 3.6 ± 2.1°, mean ME = 0.02 ± 3.75°	Marker-based Vicon motion capture system was utilized and inverse kinematics was conducted in OpenSim
Gholami et al. (2020)	Tri-axial linear acceleration	Filtered by a 4th order Butterworth low-pass filter with cut-off frequency of 6 Hz	Training/test: 0.80/0.20	LOSOCV	CNN (kernel size = 3, stride = 1)	Hip, knee, and ankle angles	RMSE, NRMSE, R^2^	Intra-participant model: *R*^2^ > 0.97, RMSE <3.4°, NRMSE <4.6%; inter-participant model: *R*^2^ > 0.78, RMSE <6.5°, NRMSE <11.1%	Marker-based Vicon motion capture system was utilized for collecting markers' trajectory and joint angles were calculated in Visual 3D (C-Motion inc.)
Wouda et al. (2018)	Relative orientation of the lower legs was input information in the first ANN; estimated joint angles and vertical accelerations were input in the ANN	Inertial data was down sampled to match the optical and vertical GRF data	Data of 10 and 14 km/h was used for training, running data at 12 km/h was used for test.	LOSOCV	ANN (two hidden layers, 250 and 100 neurons)	Vertical GRF and sagittal knee joint angles	*R*^2^, RMSE	Knee flexion/extension angles: RMSE <5°; vertical ground reaction force: RMSE <0.27 BW	Joint angles were collected with both Xsens MVN Link inertial and Vicon optical motion capture system; vertical ground reaction force was measured from an instrumented treadmill
Derie et al. (2020)	Auto-generated statistical features of 3D acceleration waveform; trial-specific features; subject-describing features	Filtered by a 2nd order band-pass Butterworth filter with cut-off frequencies of 0.8 and 45 Hz	/	LOSOCV; LOTOCV	EN, LASSO, XGB	VILR	MAE, *R*^2^, ROC	Subject-dependent XGB model: MAE = 5.39 ± 2.04 BW/s, *R*^2^ = 0.95; Subject-independent XGB model: MAE = 12.41 ± 7.90 BW/s; *R*^2^ = 0.77	GRF were measured by two built-in force platforms (2 and 1.2 m, AMTI)
Liu et al. (2020)	Tri-axial accelerometer and gyroscope data	The number of data points per sample and mean, standard deviation, median, maximum, and minimum of the acceleration and angular velocity data were extracted from each step and anthropometric features for RunNet-MLP	Training/test: 0.80/0.20	LOSOCV	Biomechanical parameter: RunNet-CNN (6 layers), RunNet-MLP (3 layers), and GBDT; running performance level: RunNet-MLP	Runners' performance level (novice, recreational and competitive), VALR, peak braking force and propulsion force, stride length, and running speed	Accuracy, confusion matrix, *R*^2^	Runners' performance level: an overall accuracy of 97.1%; biomechanical parameters: RunNet-CNN: *R*^2^ > 0.9	Biomechanical parameters were measured from an instrumental treadmill
Rapp et al. (2021)	Tri-axial accelerometer and gyroscope data	Synthetic accelerometry and gyroscope data were generated by taking numerical derivatives and adding Gaussian noise	Training/validation/test: 0.80/0.10/0.10	/	Conv1D, LSTM	Flexion/extension, abduction/adduction, internal/external rotation of hip, knee, and ankle	RMSE	Mean RMSE of flexion/extension <1.27 ± 0.38°, Mean RMSE of abduction/adduction <2.52 ± 0.98°, Mean RMSE of internal/external rotation <3.34 ± 1.02°	Marker-based Vicon motion capture system used for collecting markers' trajectory and joint angles were calculated with custom software (Running Injury Clinic Inc.)
Ngoh et al. (2018)	Acceleration along x-axis	Acceleration was filtered using 2nd Butterworth low-pass filter with cut-off frequency of 10 Hz	Training/validation/test: 280 trials for training, 120 trials for validation and testing; Remain 230 data for accuracy evaluation	/	ANN (two hidden layers, 10 and 100 neurons)	Vertical GRF	*R*^2^, RMSE	RMSE <0.017 BW, *R*^2^ > 0.99	Vertical GRF was measured from an instrumented treadmill
Young et al. (2020)	Tri-axial accelerometer and gyroscope data	Degree of pronation (neural, slight, and severe) and foot strike type (heel, midfoot, and forefoot) were measured or calculated from raw data	Training/test: 0.75/0.25	/	Ensemble deep learning model (a MLP classifier, a GB classifier, and a custom-train ANN model)	Recommending running shoes type	Accuracy	Accuracy = 94.6%	/
Robberechts et al. (2021)	Filtered acceleration, Jerk, roll, pitch, acceleration right x peak min	Filtered by a 2nd order band-pass Butterworth filter with cut-off frequencies of 0.8 and 45 Hz	The perceptron model: training/test: 83/10 subjects; The RNN model: training/validation/test: 73/10/10 subjects	5-fold CV, LOSOCV	The averaged structured perceptron algorithm; RNN (two bidirectional long short-term memory layers, 50 hidden neurons, dropout 20% after each layers)	Gait event detection (initial contact and toe off), stance time	MRE, MAE, ROC	The perceptron model: IC: MAE = 2.00 ± 2.89, TO: MAE = 9.00 ± 8.18, ST: MAE = 10.00 ± 8.73; The RNN model: IC: MAE = 2.00 ± 3.29, TO: MAE = 4.00 ± 4.52, ST: MAE = 6.50 ± 5.74	Gait event were detected by two built-in force platforms (2 and 1.2 m, AMTI)
Zrenner et al. (2018)	Tri-axial accelerometer and gyroscope data	The IMUs data in each stride was zero padded to 200 samples	/	LOSOCV	CNN (two convolutional layers, two max pooling layers, one flattening layer, two fully-connected layers, and one 30% dropout layer)	Stride length and velocity; distance of running (3.2 km)	ME, MAE, MAPE	Running stride length: ME = 2.5 ± 20.1 cm, MAE = 15.3 cm, MAPE = 5.9%; velocity: ME = 0.055 ± 0.285 m/s, MAE = 0.216 m/s, MAPE = 5.9%; distance of running: MAE = 194.5 m	The Vicon motion capture system was used as the gold standard for velocity and stride length; total distance of field running was recorded using GPS by a smartphone (Galaxy S8, Samsung Inc.)
Komaris et al. (2019)	Tri-axial linear acceleration	Data were standardized using Z-score normalization	Training/validation/ test: 0.60/0.20/0.20	LOSOCV	ANN (one hidden layer with 10 neurons)	Vertical, anterior-posterior, and medial-lateral GRF	RMSE for force-time waveform evaluation; ME for peak force evaluation	RMSE: Vertical GRF: 0.134 ± 0.027 BW, anteroposterior GRF: 0.041 ± 0.007 BW, and mediolateral GRF: 0.042 ± 0.006 BW	GRF was measured using an instrumented dual-belt treadmill (Bertec Corp.)
Tan et al. (2019)	Tri-axial linear acceleration and composite accelerations over three timesteps	Data were scaled to a range of 0–5 using Min-Max scaling	Training/validation/test: 0.47/0.23/0.30	/	LSTM (five layers, 44 hidden neurons in each layer)	Gait event detection (heel strike and toe off)	F1, Precision, Recall, and MAE	F1: heel strike: treadmill run = 0.92, indoor run = 0.96, outdoor run = 0.92; toe off: treadmill run = 0.77, indoor run = 0.86, outdoor run=0.81	/
Watari et al. (2018a)	Tri-axial pelvic acceleration, patient reported outcome measures and demographic variables	Raw data were standardized to a mean of 0 and a standard deviation of 1, dimensionality reduction was performed with PCA	/	10-fold CV	PCA (for feature extraction), SVM	Classifying patellofemoral pain cohort	Accuracy, precision, recall, F1-score, MCC, confusion matrix	Accuracy: 85.4%, precision: 90.0%, recall 96.4%, F1-score: 0.93, MCC: 0.69	/
Watari et al. (2018b)	Tri-axial pelvic acceleration	Dimensionality reduction was performed with PCA, each step was normalized to 100 points and standardized to zero mean and unit variance	/	/	PCA (for feature extraction), HCA	Clustering patellofemoral pain patients into homogeneous subgroups	/	Two subgroups were identified for female runners	/
Ahamed et al. (2019)	Pelvic drop, vertical oscillation of the pelvis, ground contact time, braking, pelvic rotation, and cadence	/	Subject-specific approach	LOSOCV	RF	Classifying inclination conditions (downhill, level, and uphill) and determining the importance of each variable	Accuracy	Subject-specific approach: mean accuracy = 86.29%; LOSOCV approach: mean accuracy = 76.17%	/
Ahamed et al. (2018)	Pelvic drop, vertical oscillation of the pelvis, ground contact time, braking, pelvic rotation, and cadence	Biomechanical variables were averaged for each ten-strides	Training/test: 0.70/0.30	One-against-another	RF (the number of trees: 100)	Classifying changes in subject-specific running gait patterns based on the environmental weather conditions and ranking the importance of biomechanical variables	Accuracy	Partitioning datasets: accuracy = 95.42%; One-against-another: accuracy = 87.18%	/
Clermont et al. (2019a)	Cadence, braking, vertical oscillation of pelvis, pelvic rotation, pelvic drop, and ground contact time	Biomechanical variables were averaged for each ten-strides	/	/	K-means clustering	Clustering running patterns throughout the marathon based on running gait alternations	/	Runners were clustered into two subgroups	/
Dixon et al. (2019)	Tri-axial linear acceleration	The first 2s of each trial were excluded, then the data were scaled from 0 to 1 according to the minimum and maximum value in the set of available trials for each subject; statistical, autocorrelation, sample entropy, smoothness, body load, and wavelet-derived energy features were extracted for the GB model	Training/test: 90%/10%	/	GB and CNN (two convolutional layers, one max pooling layer, two convolutional layers, one global average pooling layer and one drop out layer with probability of 0.5)	Classifying three different surfaces (concrete road, synthetic track, woodchip trail)	Accuracy, precision, recall, F1-score, confusion matrix	Accuracy: GB: concrete:93.7 ± 2.8, synthetic: 92.2 ± 2.1, woodchip: 95.7 ± 2.4, average: 93.9 ± 1.9; CNN: concrete:95.9 ± 4.0, synthetic: 94.7 ± 3.3, woodchip: 97.6 ± 1.2, average: 96.1 ± 2.6	/
Johnson et al. (2021)	Tri-axial linear acceleration and time	4D acceleration inputs were flattened into 2D images by representing the five sensors' locations on the horizontal axis, stance-normalized time frames upwards on the vertical axis	/	/	Two CNN models CaffeNet and ResNet-50	GRF	*R*^2^, rRMSE	For moderate speed running of the left stance limb using CaffeNet, Vertical GRF: *R*^2^ = 0.97, rRMSE = 13.93%; For slow speed running of the left stance limb using ResNet-50, anterior-posterior GRF: *r* = 0.96, rRMSE = 17.06%;	Kinematics and kinetics were recorded and calculated with Vicon optical motion capture system and AMTI force plate
Tan et al. (2020)	Tri-axial accelerometer and gyroscope	Min-max normalization was used to normalize each IMU channel	/	LOSOCV	CNN (3 hidden layers with 50, 50, and 10 neurons, respectively)	VALR	*R*^2^, MAE, NRMSE	*R*^2^ = 0.94 ± 0.03, MAE = 13.8 ± 5.8 BW/s, NRMSE = 9.7 ± 3.6%	GRF data were collected using an instrumented dual-belt treadmill (Bertec Corp.)
Koska and Maiwald (2020)	Sagittal plane (gyroscope) data	Filtered by a 4th order low-pass Butterworth filter with cut-off frequencies of 20 Hz, data were normalized between 0 and 1	10, 20, 50, and 100% dataset were used for training model, respectively	/	KNN	Classifying the subjective perception of running shoe comfort (comfortable and uncomfortable)	CCR	Mean CCR = 0.92	/
Matijevich et al. (2020)	Foot and shank minimum and maximum angles and angles at midstance	Feature were normalized to z-scores prior to model training	/	LOSOCV	LASSO	Peak force on the tibial bone	MAPE	Foot: MAPE = 7.9 ± 2.3%, shank: MAPE = 8.0 ± 2.9%	Kinetics was collected on a force-instrumented treadmill (Bertec Corp.)

*CV, cross-validation; LOSOCV, leave-one-subject-out cross-validation; LOTOCV, leave-one-trial-out cross-validation; RMSE, root-mean-squared error; rRMSE, relative root-mean-squared error; NRMSE, normalized root-mean-square error; R^2^, Pearson's correlation coefficient; ME, mean error; MAE, mean absolute error; MAPE, mean absolute percentage error; MRE, mean relative error; CCR, correct classification rate; ROC, receiver operating characteristic curves; MCC, Matthews correlation coefficient; ANN, artificial neural network; DeepConvLSTM, deep learning framework of the convolutional and LSTM recurrent neural networks; CNN, convolutional neural network; EN, linear regression with elastic net regularization; LASSO, linear regression with least absolute shrinkage and selection operator regularization; GB, gradient boosting; XGB, gradient boosted regression tree; GBDT, gradient boosting decision tree; MLP, multilayer perceptron; Conv1D, 1D convolutional neural network; LSTM, lone short-time memory; RNN, recurrent neural network; PCA, principle component analysis; SVM, support vector machine; HCA, hierarchical cluster analysis; RF, random forest; KNN, k-nearest neighbors algorithm; GRF, ground reaction force; VILR, maximal vertical instantaneous loading rate; VALR, average vertical loading rate; IMUs, inertial measurement units; BW, body weight*.

#### Predictors of Machine Learning Model

For predictors in the studies, time-series data from the trial-axis accelerometer were chosen as attributes in 15 articles (Watari et al., 2018a,b; Zrenner et al., 2018; Dixon et al., 2019; Komaris et al., 2019; Stetter et al., 2019, 2020; Tan et al., 2019, 2020; Gholami et al., 2020; Liu et al., 2020; Young et al., 2020; Hernandez et al., 2021; Johnson et al., 2021; Rapp et al., 2021). Most of the studies used deep learning neural network algorithms for prediction. Continuing trial-axis angular velocity data were considered in eight studies (Zrenner et al., 2018; Stetter et al., 2019, 2020; Liu et al., 2020; Tan et al., 2020; Young et al., 2020; Hernandez et al., 2021; Rapp et al., 2021). Sagittal plane angular velocity (Koska and Maiwald, 2020) and anterior-posterior (Ngoh et al., 2018), and vertical (Wouda et al., 2018) acceleration were investigated in three studies. Discrete biomechanical variables measured from wearable sensors were also selected as input (Watari et al., 2018a,b; Clermont et al., 2019a). Three studies extracted the statistical features in raw sensor signals as the attributes (Derie et al., 2020; Matijevich et al., 2020; Robberechts et al., 2021). The predicted outcomes of the regression algorithms were compared with the ground truth reference data, which was measured from the optical motion capture system and force plate. For the collection of continuing time-series data, the force-instrumented treadmill was employed.

#### Cross-Validation and Evaluation

Several studies had no validation process for the machine learning algorithms in the methodology section (Ahamed et al., 2018, 2019; Watari et al., 2018a; Zrenner et al., 2018; Dixon et al., 2019; Derie et al., 2020; Koska and Maiwald, 2020; Matijevich et al., 2020; Stetter et al., 2020; Tan et al., 2020; Young et al., 2020; Johnson et al., 2021). In one study, KNN was trained in different proportions of the dataset, but trained models were not validated or tested (Koska and Maiwald, 2020). Half of the studies used leave-one-subject-out cross-validation (LOSOCV) methods (Wouda et al., 2018; Zrenner et al., 2018; Ahamed et al., 2019; Komaris et al., 2019; Stetter et al., 2019, 2020; Derie et al., 2020; Gholami et al., 2020; Liu et al., 2020; Matijevich et al., 2020; Tan et al., 2020; Robberechts et al., 2021). For the regression assignments, Pearson's correlation coefficient (*R*^2^), root-mean-squared error (RMSE), mean error (ME), and mean absolute error (MAE) were utilized to assess the model's performance. Accuracy, F1 score, precision, recall, confusion matrix, Matthews correlation coefficient (MCC), and receiver operating characteristic curves (ROC) were employed to evaluate the classification problems.

## Discussion

This systematic study evaluated the use of wearable inertial sensors combined with machine learning and deep learning algorithms in the field of low limb running biomechanics. The pelvis, tibia, and foot were common locations for the sensor placement, and two sensors were most frequently adopted. Simulated IMU signals were also explored by converting marker trajectories into accelerations *via* numerical differentiation (Johnson et al., 2021; Rapp et al., 2021). It was found that the use of IMU sensors with machine learning approaches emerged recently (from 2018). The performance of assessing joint angles, forces, moments and GRF, and identifying and classifying multiple conditions were investigated. Furthermore, processing time-series data from IMUs using deep learning algorithms to predict lower limb biomechanics and classification tasks are becoming increasingly prevalent.

### Machine Learning-Based Methods for Evaluating Running Biomechanics

Musculoskeletal models and kinematic chain models are physics-based approaches introduced to calculate gait kinematics and kinetics from IMU sensors (Karatsidis et al., 2017; Picerno, 2017; Dorschky et al., 2019). Subject-specific anthropometric data, however, are mandatory to scale the musculoskeletal model (Stetter et al., 2020). This process could inevitably cause inaccuracy (Faber et al., 2016; Ancillao et al., 2018). Kinematic chain modeling needs to capture the kinematic behaviors of main body segments by attaching one sensor to each segment (Wouda et al., 2018). Therefore, it takes a longer time for the experimental setup, requires multiple sensors, and constrains gait movement (Ngoh et al., 2018). In contrast to physics-based models, data-driven approaches used fewer sensors and built-up and optimized model parameters by training the model using part of the data rather than requiring any prior knowledge of the model (Wouda et al., 2018; Stetter et al., 2020).

The accuracy of predicted knee joint angles using CNN is higher than hip and ankle angles, even though the sensor data were obtained from the foot (Gholami et al., 2020). This means the sensor location may not be the most critical factor for the estimation of lower joint kinematics. Another factor, for instance, the flexibility of joints, may also affect the accuracy. Tenforde et al. (2020) found that tibial acceleration is associated with the GRF matrix in injured runners. However, Matijevich et al. (2019, 2020) clarified that the vertical average loading rate (VALR) during running is not strongly correlated with peak tibial force. Miller et al. (2019) using tibial acceleration combined with ANN predicted vertical GRF across multiple speeds [RMSE = 0.16 body weight (BW), *R*^2^ = 0.97]. Linear regression with least absolute shrinkage and selection operator regularization (LASSO) regression algorithm also showed promising results for evaluating peak tibial bone load from pressure and foot-mounted IMU data (Matijevich et al., 2020). These findings highlight that machine learning and deep learning algorithms could successfully predict response even though the relationship between inputs and output is still unclear and these issues can be explained from the perspective of data-driven approaches.

### Model Assessment

Only two public datasets of running biomechanics (Komaris et al., 2019; Tan et al., 2019) were employed for analysis within the included studies, and the sample size is limited (below thirty). Generally, the CNN model sets 2–3 hidden layers, the LSTM usually contains two layers, and the ANN layer varies from 1 to 3 layers. The number of neurons in each hidden layer ranged from 10 to 250. Adam was the most frequently used optimizer, but those should be determined by hyperparameter tuning for the best combination (Hernandez et al., 2021). The predicted error of sagittal joint angles from the simulated IMU data has been shown to decrease (Dorschky et al., 2020). This might be due to the simulated data being smoother than the measured data (with less noise effect) (Rapp et al., 2021). Different validation or test approaches were compared to check their influence on the predicted accuracy (Wouda et al., 2018; Derie et al., 2020; Gholami et al., 2020; Liu et al., 2020). This review noticed that some studies did not validate the model's performance, which should be improved in future research. Best accuracy was achieved by splitting one subject's trials into both training and test procedures. The data not seen during testing were vital to assess machine learning models' performance and improve confidence in its practice (Halilaj et al., 2018). This intra-participant method could reduce the reliability and practicality in real-world applications (Ahamed et al., 2019; Derie et al., 2020; Gholami et al., 2020). Nested k-fold CV could conduct both hyperparameter tuning and evaluation based on inner and outer loops (Hernandez et al., 2021). It is recommended to use the LOSOCV or Nested k-fold CV method to validate or test the model's performance. The leave-one-trial-out cross-validation (LOTOCV) or random train test split approach will be only suitable for validating or testing the subject-specific machine learning model (Ahamed et al., 2019; Derie et al., 2020).

### The Practice of Deep Learning

The deep learning technique takes time-series data into the input and has high computational efficiency compared with traditional machine learning approaches. Both CNN and RNN are popular tools utilized in lower extremity running biomechanics. A new approach (Ordóñez and Roggen, 2016) called DeepConvLSTM developed from human activity recognition classification was adopted to predict lower limb kinematics and shows state-of-the-art accuracy (Hernandez et al., 2021). Spatiotemporal features of multiple wearable sensors were extracted through CNN and RNN layers in these neural networks. Ihianle et al. (2020) found that DeepConvLSTM achieved the highest accuracy for the classification of jogging and running from multiple daily activities, compared to CNN and LSTM algorithms based on wearable sensor data. However, currently, only lower limb kinematics has been estimated using the DeepConvLSTM model. This review demonstrated that running kinetics is the most investigated prediction task. Based on the knowledge gap, it could be valuable to explore this algorithm's ability to predict lower limb kinetics during running. However, the current applications of the deep learning technique suffer from one main pitfall. Data-driven deep learning algorithms require large datasets for model training, but such datasets are scarce in running biomechanics compared with gait datasets in walking, which incorporate ground truth values from the motion capture system and wearable sensor data. On the other hand, data augmentation or transfer learning techniques can be considered to improve the model's generalization and performance on the limited training dataset (Komaris et al., 2019; Rapp et al., 2021).

### Recommendations for Future Studies

According to the information from these included articles and analysis, there are several limitations in the reviewed previous studies. The following directions are identified and should be reviewed for future research regarding machine learning in running biomechanics by using wearable inertial sensor data:

- The sample size to garner sensor data for assessing running biomechanics is recommended to be larger than 20.
- One or two sensors are enough to obtain predictors inputting to the machine learning and deep learning models.
- The tibia, foot, and pelvis are frequently employed locations for sensors' attaching.
- Subject-independent (inter-participant) methods should be used to test the performance of the machine learning model (Derie et al., 2020; Liu et al., 2020; Tan et al., 2020; Hernandez et al., 2021).
- Raw acceleration data is recommended as input to capture the variabilities of spatial and temporal features (Dixon et al., 2019).
- Acceleration data are among the most commonly adopted wearable inertial data in lower extremity running biomechanics.
- Deep learning approaches could be more suitable for dealing with time-series data from wearable sensor data.
- Hyperparameter tuning is not only beneficial for picking the best combination of the model's parameters but also for the model's structure selection (Hernandez et al., 2021).
- Dividing training, validation, and testing datasets rigorously and presenting data not seen before during testing are essential to assess machine learning models' generalization and improve confidence in their practice (Halilaj et al., 2018).
- CNN, ANN, LSTM, DeepConvLSTM, MLP, and GB are popular algorithms to process IMUs data.
- The implementation of DeepConvLSTM for exploring wearable sensor signals in the field of lower limb running biomechanics is generally promising (Hernandez et al., 2021).
- Data augmentation or transfer learning approaches provide us with a novel viewpoint on running biomechanics, given the scarcity of data currently accessible in the field (Komaris et al., 2019; Rapp et al., 2021).

## Conclusion

This study reviewed the current practice and trend in the realm of lower extremity biomechanics during running. Machine learning approaches, especially deep learning approaches, have rapidly arisen in recent years due to wearable technology improvements in gait analysis. Machine learning algorithms showed state-of-the-art predictability for processing wearable inertial data. However, in the future, the validation procedure for machine learning models should receive increased emphasis. A deep learning model combining resemble CNN and RNN should be utilized to extract different running features from the IMUs sensor. Investigating both upper limb and lower limb biomechanics for future studies would be interesting and worthwhile as running is a whole-body action.

## Data Availability Statement

The original contributions presented in the study are included in the article/supplementary material, further inquiries can be directed to the corresponding authors.

## Author Contributions

LX conceived the study idea, conducted study screening, and drafted the manuscript. AW, YG, LZ, VS, and JF conceived the study idea, assisted in revising the manuscript, and reviewed the first and final versions of the manuscript. All authors contributed to the article and agreed to the submitted version of the manuscript.

## Funding

This work was supported by the National Key R&D Program of China (2018YFF0300905), the Key R&D Program of Zhejiang Province, China under Grant 2021C03130; Public Welfare Science and Technology Project of Ningbo, China under Grant 2021S133; Zhejiang Province Science Fund for Distinguished Young Scholars under Grant R22A021199; K. C. Wong Magna Fund in Ningbo University. LX would like to thank the support from China Scholarship Council (CSC).

## Conflict of Interest

The authors declare that the research was conducted in the absence of any commercial or financial relationships that could be construed as a potential conflict of interest.

## Publisher's Note

All claims expressed in this article are solely those of the authors and do not necessarily represent those of their affiliated organizations, or those of the publisher, the editors and the reviewers. Any product that may be evaluated in this article, or claim that may be made by its manufacturer, is not guaranteed or endorsed by the publisher.
